# Rare germline variants in canonical and candidate genes in apparently sporadic bilateral primary aldosteronism

**DOI:** 10.1210/jendso/bvag167

**Published:** 2026-07-27

**Authors:** Lucas S Santana, Débora K Alves-Fernandes, Jose Antonio B Lima Sobrinho, Felipe Freitas-Castro, Lucas B Rossetti, Jessica Okubo, Gustavo F C Fagundes, Eduardo Z Kawahara, Thiago S Mendes, Luiz A Bortolotto, Andrea Pio-Abreu, Giovanio V Silva, Luciano F Drager, Ana Claudia Latronico, Madson Q Almeida

**Affiliations:** Unidade de Adrenal, Laboratório de Endocrinologia Molecular e Celular LIM25, Divisão de Endocrinologia e Metabologia, Hospital das Clínicas, Faculdade de Medicina da Universidade de São Paulo, São Paulo 01246-903, Brasil; Unidade de Adrenal, Laboratório de Endocrinologia Molecular e Celular LIM25, Divisão de Endocrinologia e Metabologia, Hospital das Clínicas, Faculdade de Medicina da Universidade de São Paulo, São Paulo 01246-903, Brasil; Unidade de Adrenal, Laboratório de Endocrinologia Molecular e Celular LIM25, Divisão de Endocrinologia e Metabologia, Hospital das Clínicas, Faculdade de Medicina da Universidade de São Paulo, São Paulo 01246-903, Brasil; Unidade de Adrenal, Laboratório de Endocrinologia Molecular e Celular LIM25, Divisão de Endocrinologia e Metabologia, Hospital das Clínicas, Faculdade de Medicina da Universidade de São Paulo, São Paulo 01246-903, Brasil; Unidade de Adrenal, Laboratório de Endocrinologia Molecular e Celular LIM25, Divisão de Endocrinologia e Metabologia, Hospital das Clínicas, Faculdade de Medicina da Universidade de São Paulo, São Paulo 01246-903, Brasil; Unidade de Adrenal, Laboratório de Endocrinologia Molecular e Celular LIM25, Divisão de Endocrinologia e Metabologia, Hospital das Clínicas, Faculdade de Medicina da Universidade de São Paulo, São Paulo 01246-903, Brasil; Unidade de Adrenal, Laboratório de Endocrinologia Molecular e Celular LIM25, Divisão de Endocrinologia e Metabologia, Hospital das Clínicas, Faculdade de Medicina da Universidade de São Paulo, São Paulo 01246-903, Brasil; Unidade de Adrenal, Laboratório de Endocrinologia Molecular e Celular LIM25, Divisão de Endocrinologia e Metabologia, Hospital das Clínicas, Faculdade de Medicina da Universidade de São Paulo, São Paulo 01246-903, Brasil; Unidade de Adrenal, Laboratório de Endocrinologia Molecular e Celular LIM25, Divisão de Endocrinologia e Metabologia, Hospital das Clínicas, Faculdade de Medicina da Universidade de São Paulo, São Paulo 01246-903, Brasil; Unidade de Hipertensão, Instituto do Coração (InCor), Hospital das Clínicas, Faculdade de Medicina da Universidade de São Paulo, São Paulo 05403-900, Brasil; Unidade de Hipertensão, Disciplina de Nefrologia, Hospital das Clínicas, Faculdade de Medicina da Universidade de São Paulo, São Paulo 05403-000, Brasil; Unidade de Hipertensão, Disciplina de Nefrologia, Hospital das Clínicas, Faculdade de Medicina da Universidade de São Paulo, São Paulo 05403-000, Brasil; Unidade de Hipertensão, Instituto do Coração (InCor), Hospital das Clínicas, Faculdade de Medicina da Universidade de São Paulo, São Paulo 05403-900, Brasil; Unidade de Hipertensão, Disciplina de Nefrologia, Hospital das Clínicas, Faculdade de Medicina da Universidade de São Paulo, São Paulo 05403-000, Brasil; Unidade de Adrenal, Laboratório de Endocrinologia Molecular e Celular LIM25, Divisão de Endocrinologia e Metabologia, Hospital das Clínicas, Faculdade de Medicina da Universidade de São Paulo, São Paulo 01246-903, Brasil; Unidade de Adrenal, Laboratório de Endocrinologia Molecular e Celular LIM25, Divisão de Endocrinologia e Metabologia, Hospital das Clínicas, Faculdade de Medicina da Universidade de São Paulo, São Paulo 01246-903, Brasil; Unidade de Oncologia Endócrina, Instituto do Câncer do Estado de São Paulo (ICESP), Faculdade de Medicina da Universidade de São Paulo, São Paulo 01246-000, Brasil

**Keywords:** bilateral primary aldosteronism, genetics, exome sequencing

## Abstract

**Background:**

The germline genetic basis of bilateral primary aldosteronism (PA) remains poorly understood, particularly in apparently sporadic disease. We investigated rare germline variants in canonical and candidate genes in patients with bilateral PA associated with resistant hypertension and/or hypertension diagnosed before 40 years of age.

**Methods:**

We studied 53 individuals with bilateral PA after exclusion of familial PA types 1 and 3. Whole-exome sequencing was performed in 52 probands and 1 affected relative. Variant prioritization was restricted to genes with direct or indirect evidence linking them to PA using a virtual panel approach. Clinical, biochemical, and imaging data were compared between carriers and noncarriers.

**Results:**

Nine of 53 patients (17%) harbored at least one rare germline candidate variant. Variants in canonical PA genes were identified in 5 patients (9.6%), including *CACNA1H* (*n* = 4) and *CACNA1D* (*n* = 1). In 4 patients (7.5%), rare variants were identified in plausible candidate genes (*ANO1, KCNK3, CASZ1,* and *STRN*). All prioritized variants were classified as variants of uncertain significance. Compared with noncarriers, carriers showed a lower frequency of hypokalemia (*P* < .0001), higher prevalence of family history of hypertension (*P* < .0001), higher frequency of resistant hypertension (*P* < .0001), required more antihypertensives (*P* = .0264), and had more frequent adrenal computed tomography abnormalities, particularly bilateral involvement (*P* < .0001).

**Conclusion:**

Rare germline variants in canonical and candidate genes were identified in a subset of patients with apparently sporadic bilateral PA. Variants in established genes, particularly *CACNA1H*, were relatively frequent, with additional variants in biologically plausible candidates.

Arterial hypertension is one of the leading risk factors for premature mortality, affecting approximately 10-40% of the global population (1, 2). Primary aldosteronism (PA) represents the most common cause of endocrine hypertension, with an estimated prevalence of about 4% among patients treated in primary care settings and up to 10% in tertiary care centers, reaching nearly 20% among individuals with resistant hypertension (3-6).

PA is characterized by the autonomous secretion of aldosterone, independent of the renin–angiotensin system. Consequently, sodium retention, suppression of plasma renin activity, elevation of blood pressure, and increased potassium excretion occur, leading to cardiovascular damage (7). The most recent Endocrine Society clinical guideline recommends screening for primary aldosteronism in all individuals with hypertension, taking into account local capacity to perform such screening (8).

Aldosterone is a mineralocorticoid hormone synthesized by the zona glomerulosa of the adrenal cortex. It plays a major role in electrolyte homeostasis by promoting renal sodium and water reabsorption (9, 10). The effects of aldosterone are mediated by the mineralocorticoid receptor, a ligand-activated nuclear receptor expressed in epithelial tissues, such as the distal renal tubule and salivary glands, as well as in non-epithelial tissues including the heart and blood vessels (9).

PA can be caused by bilateral aldosterone excess (idiopathic hyperaldosteronism) or unilateral disease (11). Bilateral and unilateral PA account for approximately 50-60% and 40-50% of PA cases, respectively (7, 11), with approximately 5% of bilateral PA occurring in a familial context. Recently, the use of aldosterone synthase (CYP11B2) immunostaining has advanced the identification of the source of aldosterone production. According to the recent international histopathology consensus (HISTALDO), which incorporates hematoxylin-eosin (H&E) and CYP11B2 immunostaining, aldosterone-producing lesions are classified into the following subtypes (12): (1) Aldosterone-producing adenoma (APA); (2) aldosterone-producing nodule (APN); (3) aldosterone-producing micronodule (APM); and (4) aldosterone-producing diffuse hyperplasia. Aldosterone-producing lesions are further categorized into classical and non-classical histology, a distinction with prognostic implications after unilateral adrenalectomy (12, 13). The classical subtype is characterized by a solitary APA or APN, whereas the non-classical subtype includes adrenals with multiple APNs, multiple APMs, a combination of both, or diffuse aldosterone-producing hyperplasia. Unilateral PA is commonly associated with classical histology, whereas bilateral PA is usually related to non-classical forms, as observed in patients undergoing unilateral adrenalectomy (13, 14). While somatic variants are identified in approximately 90% of aldosterone-producing lesions when guided by CYP11B2 immunostaining, limited progress has been made in elucidating the germline genetic basis of bilateral PA (9, 15-18).

Several genes encoding ion channels that regulate zona glomerulosa cell depolarization and aldosterone biosynthetic pathways have been implicated in the pathogenesis of familial PA, including the *CYP11B1/CYP11B2* chimeric gene (19), *KCNJ5* (20), *CACNA1D* (21), *CACNA1H* (22), and *CLCN2* (23). However, germline variants associated with bilateral disease remain underdiagnosed due to the lack of routine genetic screening. Consequently, epidemiological data on the prevalence of these genetic causes remain limited (24).

This study seeks to broaden the spectrum of genetic etiologies underlying bilateral PA. Germline whole-exome sequencing (WES) was conducted in 54 patients with bilateral PA associated with resistant hypertension and/or diagnosis before age 40 who were negative for the *CYP11B1/CYP11B2* chimeric gene and germline *KCNJ5* pathogenic variants in prior targeted screening.

## Patients and methods

This study was conducted in accordance with the ethical principles outlined in the Declaration of Helsinki and was approved by the Ethics Committee of the Clinics Hospital, University of São Paulo Medical School (approval number #7.522.390). Written informed consent was obtained from all participants or their legal guardians. Clinical, biochemical, and imaging data were collected from medical records. Probands with a clinical diagnosis of PA were recruited from the Endocrine Hypertension Outpatient Clinic of the Division of Endocrinology and Metabolism, the Secondary Hypertension Outpatient Clinic of the Division of Nephrology, and the Hypertension Outpatient Clinic of the Heart Institute (InCor) of the Clinics Hospital, University of São Paulo Medical School (FMUSP).

### Investigation of PA

A positive screening for PA was defined as an aldosterone-to-direct renin concentration (A/DRC) ratio greater than 2 ng/dL/µIU/mL (equivalent to 2 ng/dL/mIU/L or 55.5 pmol/L/µIU/mL), in the presence of concomitant aldosterone levels exceeding 10 ng/dL (277 pmol/L). Suppressed DRC was defined as a direct renin concentration below 4 mIU/mL. To account for intraindividual variability in aldosterone secretion, PA screening was repeated at least twice (25). Serum aldosterone and plasma DRC were quantified using an automated chemiluminescent immunoassay (LIAISON kit, DiaSorin, Saluggia, Italy) in all patients, as previously described (26). Management of antihypertensive medications for PA diagnosis was conducted according to established protocols (25, 26).

After biochemical confirmation of PA, patients underwent adrenal computed tomography (CT) for etiological assessment and to exclude adrenal cortical carcinoma. However, adrenal CT presents limited accuracy (approximately 60-70%), particularly for detecting small (<1 cm) aldosterone-producing lesions (7) or in patients with bilateral adrenal nodules, where distinguishing non-functioning or cortisol-secreting adenomas may be challenging. Sequential adrenal venous sampling (AVS) was performed after biochemical and CT evaluation, as previously described (27, 28). AVS was not indicated for patients younger than 35 years at the time of hypertension diagnosis, especially in those presenting with spontaneous hypokalemia and markedly elevated aldosterone levels (7, 8, 29).

Inclusion criteria were (1) bilateral PA confirmed by AVS under cosyntropin stimulation (lateralization index <4), or by the absence of biochemical cure after unilateral adrenalectomy in patients with CYP11B2-positive lesions (APNs or APMs) identified in the resected adrenal gland and (2) resistant hypertension and/or diagnosis of hypertension before 40 years of age, with prior exclusion of familial PA type 1 (glucocorticoid-remediable aldosteronism due to the *CYP11B1/CYP11B2* chimeric gene) and type 3 (germline pathogenic variants in *KCNJ5*). The clinical and biochemical characteristics of the cohort are summarized in Table 1, and individual-level data are provided in Table S1 (30).

**Table 1:** Clinical, biochemical, and imaging characteristics of carriers and noncarriers of germline candidate variants

Cohort characteristics	Total patients	Variant carriers (*n* = 9)	Noncarriers (*n* = 44)	*P*-value
**Sex, *n* (%)**				.8759
** Female**	34 (64.2)	6 (66.6)	28 (63.6)	
** Male**	19 (35.8)	3 (33.3)	16 (36.4)	
**Resistant hypertension** *^b^* **n (%)**	44 (84.6)	9 (100)	35 (81.4)	<0,0001
**Number of antihypertensive drugs** *^a^*	4 (3.0, 5.5)	7 (4.5, 7.0)	4 (3.0, 5.0)	.0264
**Surgical intervention*^c^* n (%)**	6 (11.3)	0 (0)	6 (13.6)	.0001
**Adrenal vein sampling, AVS (%)**	52 (98.1)	9 (100)	43 (97.7)	.1552
**Age at diagnosis of HT (years)** *^a^*	34 (26.0, 39.25)	30 (27.0, 44.0)	34 (25.5, 39.0)	.7187
**Age atdiagnosis of PA (years)** *^a^*	54 (43.5, 58.0)	55 (46.0, 63.0)	54 (41.25, 58.0)	.4942
**Potassium (mEq/L)** *^a^*	4.2 (3.9, 4.45)	4.3 (4.0, 4.45)	4.1 (3.825, 4.475)	.6032
**Aldosterone (ng/dL)** *^a^*	21.3 (16.7, 29.1)	20.2 (15.6, 24.7)	22.0 (16.7, 29.7)	.3328
**Renin (μU/mL)** *^a^*	4.0 (4.0, 4.0)	4.0 (4.0, 5.55)	4.0 (4.0, 4.0)	.3861
**Cortisol laterization index** *^a^*	1.53 (1.205, 2.26)	1.53 (1.185, 2.55)	1.495 (1.203, 2.233)	.6286
**Metanephrine lateralization index** *^a^*	1.745 (1.275, 2.4425)	1.740 (1.225, 4.4)	1.75 (1.305, 3.2)	.9396
**Hypokalemia, *n* (%)**				<.0001
** Yes**	18 (34.6)	1 (11)	17 (39.5)	
** No**	34 (65.4)	8 (89)	26 (60.4)	
**Computed tomography, *n* (%)**				.0015
** Normal**	21 (39.6)	2 (22.2)	19 (43.2)	
** Abnormal (unilateral and bilateral lesion)**	32 (60.4)	7 (77.8)	25 (56.8)	
**Laterality of the abnormality (%)**				<.0001
** Unilateral**	18 (56.2)	2 (28.6)	16 (64.0)	
** Bilateral**	14 (43.8)	5 (71.4)	9 (36.0)	
**Family history of HT, *n* (%)**				<.0001
** Present**	38 (80.9)	8 (100)	30 (76.9)	
** Absent**	9 (19.1)	0 (0)	9 (23.1)	

Reference range: serum potassium 3.0-5.3 mEq/L.

Abbreviations: HT, arterial hypertension; PA, primary aldosteronism.

^*a*^Median (IQR 25, 75); IQR, interquartile range. Continuous variables are presented as median (interquartile range), while categorical variables are expressed as absolute numbers and percentages. The number of patients may vary across variables due to missing data.

^*b*^Resistant hypertension refers to uncontrolled HT (≥140/90 mmHg) despite the use of ≥3 antihypertensive medications, or controlled HT (<140/90 mmHg) requiring ≥4 medications.

^*c*^Laparoscopic unilateral adrenalectomy or unilateral + contralateral subtotal adrenalectomy.

### Genetic investigation

All probands had previously undergone Sanger sequencing to investigate the presence of germline pathogenic variants in the canonical gene most frequently associated with the phenotype, *KCNJ5* (MIM 600734) (24). In addition, all cases were tested using long-range PCR for glucocorticoid-remediable PA, caused by the chimeric *CYP11B1/CYP11B2* gene (MIM #103900), as previously described (31).

Finally, 52 probands and 1 family member with bilateral PA and without germline pathogenic variants identified in previous targeted screening were selected for WES analysis. WES was performed using a DNA nanoball sequencing platform (DNBSEQ, BGI Genomics Europe, Herlev, Denmark) following germline exome enrichment with KAPA HyperExome Probes (Roche, CA, USA), ensuring high-quality sequencing with low duplication rates and deep coverage (32). Bioinformatic analyses were conducted using in-house pipelines for data quality control, alignment to the human reference genome, and variant calling (SNVs, small indels, and CNVs), as previously described (32). Gene and variant annotation, filtering, and prioritization were performed using Franklin (Genoox, Israel).

In silico prediction was incorporated at 2 complementary levels. Missense variant pathogenicity was assessed using REVEL (33), as implemented in the Franklin platform (Genoox, Israel). Because REVEL estimates pathogenicity independently of the direction of the functional effect, the predicted functional consequence (gain of function [GoF] or loss of function [LoF]) of missense variants occurring in voltage-gated sodium and calcium channel genes (*SCNxA* and *CACNA1x* families) was additionally evaluated using funNCion, a machine-learning tool developed for this ion channel superfamily (34). The directional prediction was considered informative only for variants classified by the tool as functionally pathogenic. For variants predicted to be functionally neutral, the directional output was considered unreliable, as indicated by the tool itself.

Table S2 summarizes the sequencing metrics, including target coverage, mean coverage depth, and the proportion of the target region covered at >10× and >20× read depth (30). All sequenced samples demonstrated high vertical and horizontal coverage. Coverage metrics were calculated using the coding sequence regions of human genes (hg19) annotated in the RefSeq database (NCBI).

**Figure 1:**
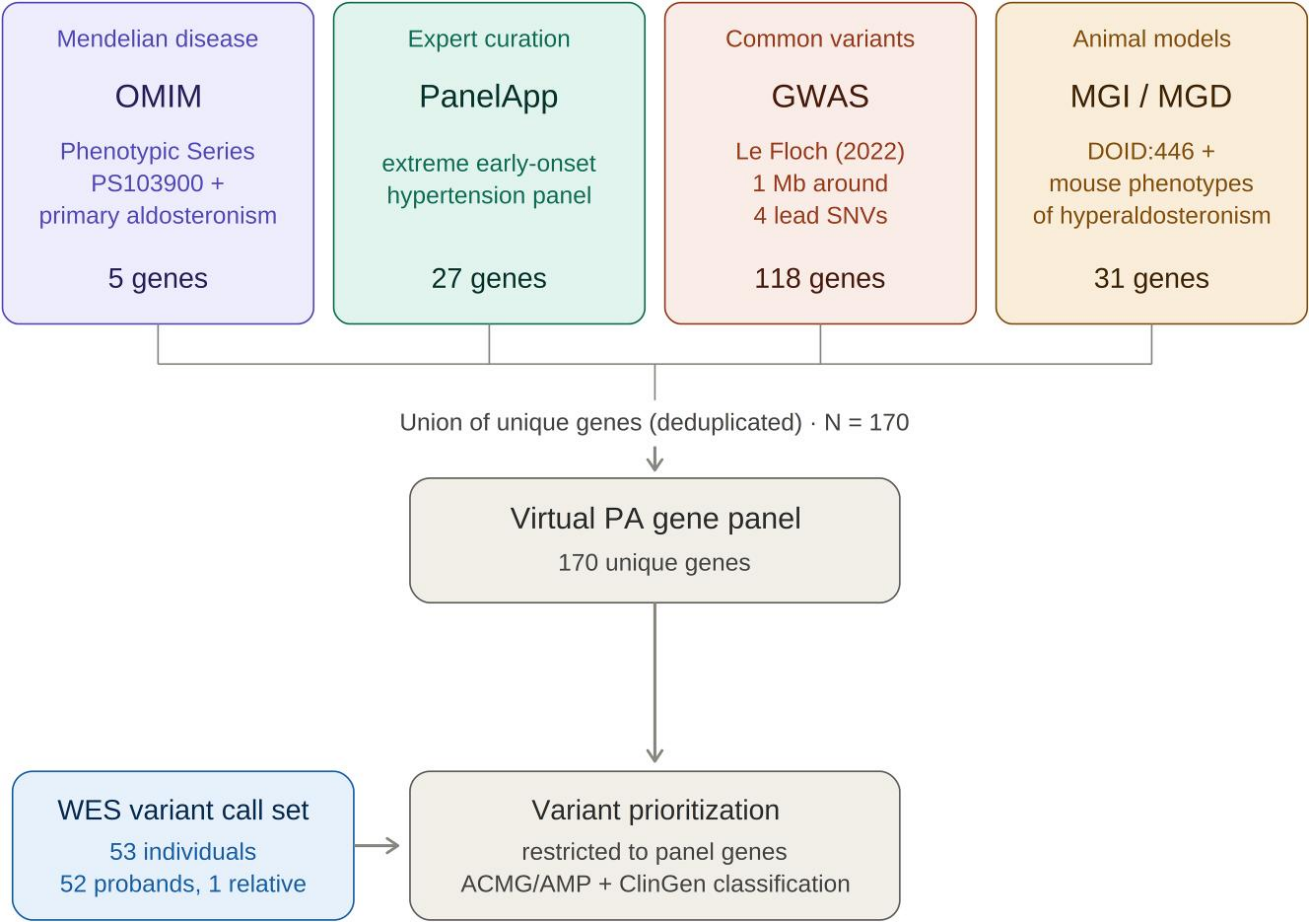
Gene enrichment approach used to construct and apply the virtual primary aldosteronism gene panel. Genes were selected from 4 independent resources, each representing a distinct layer of evidence: (1) OMIM, combining the Phenotypic Series PS103900 (Hyperaldosteronism, familial) with an OMIM term search for “primary aldosteronism,” which additionally retrieved *CACNA1D* (PASNA, MIM #615474) (5 genes); (2) the Genomics England PanelApp “Extreme Early-Onset Hypertension” panel (27 genes); (3) a meta-analysis of GWAS risk loci for primary aldosteronism, comprising all annotated genes located within a 1 Mb window flanking each of the 4 genome-wide significant lead variants (118 genes); and (4) the Mouse Genome Informatics/Mouse Genome Database (MGI/MGD), using the Disease Ontology Identifier DOID:446 (primary hyperaldosteronism) and mouse phenotypes of human hyperaldosteronism, mapped to human orthologs (31 genes). The 4 lists were merged by their union, after removal of duplicates, into a virtual panel of 170 unique genes, which was applied as a biological-plausibility filter.

Given the large number of variants identified per sample, particularly SNVs, which precluded detailed manual curation, a filtering strategy was applied based on the expected biological plausibility for the investigated phenotype. To refine the genomic target while minimizing pre-selection bias toward a single source of evidence, the analysis was restricted to genes with direct or indirect support for involvement in PA pathophysiology, assembled into a virtual gene panel through the convergent integration of 4 methodologically distinct resources, each capturing a different layer of evidence: Mendelian disease curation, expert consensus, human population-level association, and experimental animal modeling (Fig. 1; Table S3) (30).

First, the OMIM Phenotypic Series PS103900 (hyperaldosteronism) and descriptor “primary aldosteronism” was used to retrieve genes with established Mendelian causation of PA (5 genes). Second, the Genomics England PanelApp “Extreme Early-Onset Hypertension” virtual panel (version 1.21) (35) was incorporated as an expert-curated gene list designed for diagnostic interpretation in patients with severe and/or early-onset hypertension, a clinical context that substantially overlaps with that of our cohort (27 genes). PanelApp ratings reflect evidence-based consensus across multiple curators, with criteria that are independent of the present cohort. Third, to capture genes implicated by common-variant susceptibility, we incorporated loci identified in a recent meta-analysis of genome-wide association studies for PA reported by Le Floch et al (36). From this study, we considered all annotated genes located within a 1 Mb window flanking each side of the 4 significant lead variants (rs284277 on chr1:9 790 797–11 790 797; rs2137320 on chr11:884 342–2 884 342; rs1535532 on chr13:31 114 398–33 114 398; and rs5905587 on chrX:42 833 996–44 833 996), based on the commonly applied rationale for considering nearby positional candidates in GWAS mapping (118 genes) (37, 38). Fourth, to incorporate cross-species evidence of relevance for aldosterone homeostasis, we queried the Mouse Genome Informatics/Mouse Genome Database (MGI/MGD) (39) using the Disease Ontology descriptor “primary hyperaldosteronism” (DOID:446) and the “hyperaldosteronism” term in the “Mouse phenotypes & mouse models of human disease” section (31 genes). This step allowed inclusion of genes whose perturbation in murine models produces an aldosterone-related phenotype, providing functional evidence of mechanistic plausibility.

The 4 resulting gene lists were combined by their union, after removing duplicates, to define the final virtual panel (170 genes) that was applied to this WES dataset. We emphasize that each source was queried a priori and not after inspection of patient-level data. The complete list of genes retrieved from each source is provided in Table S3 (30). Genes meeting any of the above criteria were retained as the target for variant prioritization. Variants in genes outside this panel were not interpreted, consistent with the goal of restricting analysis to loci with prior biological plausibility for PA.

Variant prioritization was performed after WES and standard filtering using a predefined virtual gene panel. Importantly, this strategy was based exclusively on genetic and biological criteria and was not guided by clinical characteristics, including family history of hypertension, hypokalemia, age at diagnosis, or disease severity. Phenotypic features were therefore not used as selection criteria during variant identification.

To quantitatively assess the pathogenicity of candidate variants, the following guidelines were applied: The American College of Medical Genetics and Genomics and the Association for Molecular Pathology standards and guidelines (40); ClinGen Variant Classification Guidance (41) and The American College of Medical Genetics and Genomics and the Clinical Genome Resource (ClinGen) technical standards for the interpretation and reporting of constitutional copy-number variants (42).

For prioritized variants located in non-coding regulatory regions (eg, 5′/3′ untranslated regions, promoters, and other regulatory elements), position-specific regulatory potential was assessed using RegulomeDB v2.2 (43, 44). RegulomeDB intersects the genomic coordinates of a query variant with functional genomics data, including transcription factor (TF) ChIP-seq, chromatin accessibility (DNase-seq and ATAC-seq), chromatin-state, TF sequence motifs/computational footprints, and expression/chromatin-accessibility quantitative trait loci (eQTL/caQTL) from ENCODE, GTEx, and additional public resources. Where available, epigenomic tracks (TF and histone ChIP-seq, chromatin accessibility, and chromatin states) were filtered for adrenal gland tissue to evaluate regulatory activity in the disease-relevant context. The supporting evidence is summarized as a categorical rank and a complementary probabilistic score. These analyses were considered hypothesis generating and were not used to assign pathogenicity under ACMG/AMP criteria.

### Publicly available adrenal gene expression data

Publicly available transcriptomic data from normal human adrenal tissue were retrieved from the GTEx Portal to evaluate the expression profile of prioritized candidate genes (45). Median transcript abundance values reported as transcripts per million (TPM) were extracted and summarized descriptively.

### Statistical analysis

To characterize the study population, categorical variables were described as frequencies and percentages, while continuous variables were summarized as means and standard deviations (SD) or medians and ranges. Quantitative variables were compared between 2 groups using Student's *t*-test for normally distributed data or the Mann–Whitney *U* test for non-normal distribution. The Shapiro–Francia test was applied to evaluate normality assumption. Patients were stratified according to the presence of rare germline variants. For comparisons of categorical variables between groups, Pearson's chi-square test or Fisher's exact test was used as appropriate. All hypotheses were 2-sided and tested at a 5% significance level, with a *P*-value <.05 considered statistically significant. Calculations were performed using SPSS software (Version 25.0; SPSS Inc., Chicago, IL, USA).

## Results

### Clinical characterization

Fifty-two probands and one relative with previously negative genetic analyses were selected for WES. Among them, 44 presented with resistant hypertension, and in 41 cases the diagnosis of hypertension was established before 40 years of age. The cohort comprised 64.2% females (34 of 53) (Table 1).

In this cohort, hypokalemia was documented in 18 of 52 cases (34.6%). Most individuals required multiple antihypertensive agents, consistent with the high prevalence of resistant hypertension (84.6%). A family history of hypertension was reported in 38 of 47 cases (80.9%), and 34.2% of these had hypertension onset before 40 years of age. Because this cohort was enriched for early-onset and/or resistant hypertension undergoing genetic investigation for PA, this frequency should be interpreted cautiously and not taken as evidence of familial aggregation on its own.

At baseline, CT revealed normal adrenal glands in 21 of 53 patients (39.6%), unilateral adrenal abnormalities in 18 (56.2%), and bilateral abnormalities in 14 (43.8%). Importantly, nearly all cases (52 of 53; 98.1%) underwent AVS, which confirmed bilateral aldosterone secretion in 92.4%. Two patients showed unilateral disease at AVS (IDs 11 and 32); however, PA was not biochemically cured after unilateral adrenalectomy, and histopathological analysis of the resected adrenal glands revealed CYP11B2-positive aldosterone-producing lesions, supporting the diagnosis of bilateral disease.

On CT, adrenal imaging was normal in 21 of 53 patients (39.6%), whereas abnormalities (unilateral or bilateral lesions) were identified in the remaining 32 (60.4%). Among these 32 patients with abnormal findings, the lesion was unilateral in 18 (56.2%) and bilateral in 14 (43.8%) (Table 1).

In total, 6 patients underwent surgical intervention (5 unilateral adrenalectomies and one unilateral adrenalectomy combined with subtotal contralateral adrenalectomy). The indications for surgery included refractory hypertension with persistently suppressed renin levels (*n* = 4) and unilateral disease identified by AVS but without biochemical cure after surgery (*n* = 2). Histopathological analysis was available for all 6 operated patients, all of whom belonged to the group without prioritized genetic variants identified in this study. Histological examination revealed exclusively non-classical histology, characterized by APMs in 5 cases and multiple APNs in one case. Although surgical intervention was significantly more frequent among noncarriers (*P* = .0001), the small number of operated cases and the lack of histological data from variant carriers preclude any conclusions about associations between genetic findings and histopathological patterns.

### Genetic analysis

After applying the prioritization criteria using the virtual panel approach described above, 9 out of 53 patients (17%) had at least one germline candidate variant, with one patient harboring 2 candidate variants in different genes (proband 4). In 5 patients (9.6%), the variant was identified in a canonical gene associated with PA, whereas in the remaining 4 cases (7.5%), 5 possible novel candidate genes were prioritized (Tables 2 and 3).

**Table 2:** Candidate germline variants identified in probands with bilateral primary aldosteronism

Proband	Gene (OMIM) domain*^a^*	Variant*^b^*	PopMax*^c^* (HT | HO)	OMIM phenotype	ACMG*^d,e^*	References/evidence
**Canonical genes**						
13	*CACNA1H* (*607904) IT	c.5020C > T p.(Arg1674Cys)	0.001% (23|0)^a^	#617027—FH-IV	VUS-Cool	ClinVar ID: 460136; dbSNP: rs771932368
54		c.4657G > A p.(Val1553Ile)	0.008% (36|0)^a^		VUS-Cool	ClinVar ID: 644166; dbSNP: rs759669950
43		c.926G > A p.(Arg309His)	0.06% (68|0)^a^		VUS-Cool	ClinVar ID: 460194; dbSNP: rs201651793
42	*CACNA1H* (*607904) NA	c.1835A > C p.(Asn612Thr)	0.02% (27|0)^a^		VUS-Cool	ClinVar ID: 1044825; dbSNP: rs773099504
55	*CACNA1D* (*114206) CAC1F_C	c.5842C > G p.(Leu1948Val)	Absent	#615474—PASNA	VUS-Cool	dbSNP: rs1362992098
**Candidate genes**						
44	*ANO1* (*610108) anoctamin	c.2500A > G p.(Thr834Ala)	0.0001% (2|0)^a^	#620045 | #620687	VUS-Cool	(46, 47) MGI: 5569648^d^
38	*KCNK3* (*603220) regulatory region	c.−17G > A p.?	0.002% (4|0)^a^	#615344	VUS-Cool	(48)
49		c.−75G > T p.?	Absent		VUS-Cool	
4	*CASZ1* (*609895) NA	c.1912G > C p.(Asp638His)	Absent	NR	VUS-Cold	(49, 50)
	*STRN* (*614765) NA	c.945G > T p.(Gln315His)	Absent	NR	VUS-Cold	(51, 52); dbSNP: rs1256684598; MGI: J:232621^c^

Abbreviations: FH-IV, Familial Hyperaldosteronism type 4; MGI, Mouse Genome Informatics; NA, not annotated (protein domain); NR, not reported (phenotype); PASNA, Primary Aldosteronism, Seizures, and Neurologic Abnormalities: ^c^heterozygous loss-of-function model; ^d^conditional homozygous knockout (renal-specific deletion driven by Cdh16-Cre expression).

^*a*^Protein domain (DIOPT—ortholog mapping online resource | Harvard Medical School): IT—ion_trans domain; CAC1F_C—voltage-gated calcium channel subunit alpha, C-term; Anoctamin—calcium-activated chloride channel.

^*b*^Reference transcript RefSeq: NM_021098.3 (*CACNA1H*); NM_000720.4 (*CACNA1D*); NM_018043.7 (*ANO1*); NM_002246.3 (*KCNK3*); NM_001079843.3 (*CASZ1*); NM_003162.4 (*STRN*).

^*c*^Maximum allele frequency: ^a^gnomAD (v4.1.0); ^b^ABraOM; GME Variome; Iranome; ToMMo-4.7KJPN; GenomeAsia.

^*d*^ACMG/AMP classification: B (Benign), LB (Likely Benign), P (Pathogenic), LP (Likely Pathogenic), VUS (Variant of Uncertain Significance).

^*e*^ACGS (Association for Clinical Genomic Science) VUS/SI temperature scale: Ice Cold, Cold, Cool, Tepid, Warm, Hot.

**Table 3:** Clinical, biochemical, and genetic characteristics of probands with candidate variants

ID	Gene/Variant	Age at diagnosis (years)	Resistant HT*^a^*	Number of antihypertensive drugs (first visit)	Hypokalemia	Family history of HT	Surgical intervention*^b^*	Cortisol laterization index	Metanephrine laterization index
**Canonical genes**									
13	*CACNA1H*/c.5020C > T p.(Arg1674Cys)	30	Yes	4	Present	Present	No	1.12	NA
54	*CACNA1H*/c.4657G > A p.(Val1553Ile)	48	Yes	NA	Absent	Present	No	1.53	NA
43	*CACNA1H*/c.926G > A p.(Arg309His)	27	Yes	5	Absent	Present	No	2.63	1.22
42	*CACNA1H*/c.1835A > C p.(Asn612Thr)	27	Yes	7	Absent	Present (<40y)	No	2.88	2.13
55	*CACNA1D*/c.5842C > G p.(Leu1948Val)	48	Yes	NA	Absent	Present	No	2.47	NA
**Candidate genes**									
44	*ANO1*/c.2500A > G p.(Thr834Ala)	30	Yes	NA	Absent	Present	No	1.22	1.23
38	*KCNK3*/c.− 17G > A p.?	19	Yes	7	Absent	Present (<40y)	No	1.38	1.74
49	*KCNK3*/c.−75G > T p.?	40	Yes	7	Absent	Present (<40y)	No	1.15	4.4
4	*CASZ1*/c.1912G > C p.(Asp638His)	36	Yes	NA	Absent	Present	No	2.23	NA
	*STRN*/c.945G > T p.(Gln315His)								

Reference range: serum potassium 3.5-5.0 mEq/L.

Abbreviations: HT, arterial hypertension; NA, not available.

^*a*^Resistant hypertension refers to uncontrolled HT (≥140/90 mmHg) despite the use of ≥3 antihypertensive agents, or controlled HT (<140/90 mmHg) requiring ≥4 medications.

^*b*^Laparoscopic adrenalectomy.

Among canonical genes previously implicated in PA, rare heterozygous germline variants were detected in *CACNA1H* [4 probands—c.5020C > T p.(Arg1674Cys), c.1835A > C p.(Asn612Thr), c.926G > A p.(Arg309His), c.4657G > A p.(V al1553Ile)] and *CACNA1D* [one proband—c.5842C > G p.(Leu1948Val)] (Table 2). The variants were distributed across different functional domains, including 3 located in the ion transport domain of *CACNA1H*. To evaluate the predicted functional directionality of these variants, we applied funNCion (34). Among the 5 *CACNA1x* variants, only the *CACNA1H* p.(Val1553Ile) variant was predicted to be pathogenic and GoF, whereas the remaining variants were predicted to be functionally neutral (Table S4) (30). All 5 *CACNA1x* variants were classified as variants of uncertain significance (VUS) according to ACMG/AMP criteria.

The age at diagnosis in this group ranged from 27 to 48 years, and all exhibited resistant hypertension. Most probands required between 4 and 7 antihypertensive medications (Table 3). Hypokalemia was documented in one (*CACNA1H—*proband 13) of the 5 individuals in this group, while a positive family history of hypertension before the age of 40 years was reported in one case (proband 42). No patient in the canonical gene group underwent surgical intervention (*P* = .0001). Proband 55, who carries a germline VUS in *CACNA1D*, did not exhibit clinical features suggestive of central nervous system involvement.

In the candidate gene group, rare heterozygous germline variants were observed in *Anoctamin 1* [*ANO1—*c.2500A > G p.(Thr834Ala)], *Potassium two pore domain channel subfamily K member 3* (*KCNK3—*c.-17G > A p.? and c.-75G > T p.?), *Castor zinc finger 1* [*CASZ1—*c.1912G > C p.(Asp638His)], and *Striatin* [*STRN—*c.945G > T p.(Gln315His)], with absent or extremely low allele frequencies in population databases and classified as VUS according to ACMG/AMP criteria (Table 2). Overall, 5 variants were identified in 4 individuals with PA, representing 7.5% (4 of 53) of the cohort. The age at diagnosis ranged from 19 to 40 years, and all presented with resistant hypertension. These individuals required 7 antihypertensive drugs, and hypokalemia was absent in all cases. A positive family history of early-onset hypertension (<40 years) was observed in 2 probands (probands 38 and 49) (Table 3). None of the individuals in the candidate-gene group had undergone surgical intervention (*P* = .0001).

**Figure 2:**
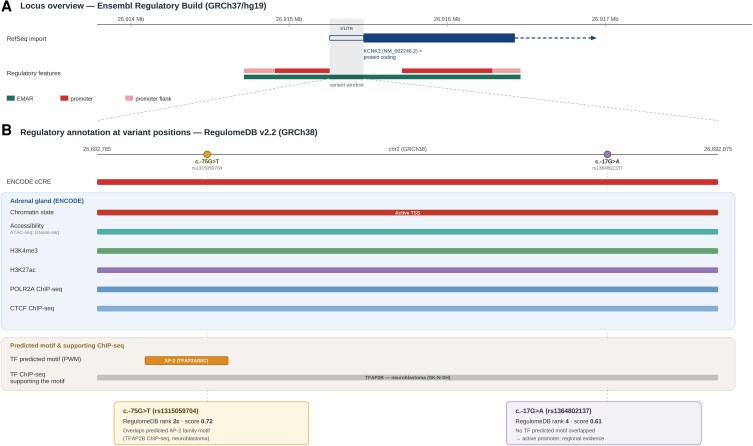
Regulatory annotation of the upstream region of the KCNK3 gene. (A) Genomic representation of the upstream region of the KCNK3 gene (GRCh37/hg19), showing regulatory elements annotated in the Ensembl Regulatory Build (version 114). A regulatory “island” is observed within the 5′ region of the canonical KCNK3 transcript, comprising promoter regions (red), promoter-flanking regions (light red), and epigenetically modified accessible regions (EMARs; dark green). The variants identified in cases 38 and 49 map to this region (shaded area corresponding to the 5′ untranslated region of exon 1), which is expanded in panel B. (B) Position-level regulatory annotation of the 2 variants, *KCNK3* c.-75G > T (rs1315059704; chr2:26 692 801) and c.-17G > A (rs1364802137; chr2:26 692 859), retrieved from RegulomeDB v2.2 (GRCh38) (PMID: 22955989; 37173523). Both variants mapped to the same ENCODE candidate cis-regulatory element (cCRE), annotated as an active transcription start site (Active TSS) in adrenal gland datasets, with supporting evidence of chromatin accessibility (ATAC-seq and DNase-seq), active promoter-associated histone marks (H3K4me3 and H3K27ac), and occupancy by RNA polymerase II (POLR2A) and CCCTC-binding factor (CTCF). The c.-75G > T variant (RegulomeDB rank 2c; score 0.72) overlapped a predicted activator protein 2 (AP-2) family transcription factor biding motif (TFAP2A/B/C), with TFAP2B ChIP-seq occupancy observed at this locus in the neuroblastoma cell line SK-N-SH; although direct adrenal TFAP2B binding data were unavailable, adrenal POLR2A and CTCF occupancy together with active chromatin marks supported the regulatory activity of this promoter element. By contrast, the c.-17G > A variant (RegulomeDB rank 4; score 0.61) was located within the same active promoter element but did not overlap any predicted transcription factor biding motif. Panels A and B are shown in different genome assemblies (GRCh37/hg19 and GRCh38, respectively). Abbreviations: AP-2, activator protein 2; cCRE, candidate cis-regulatory element; CTCF, CCCTC-binding factor; EMAR, epigenetically modified accessible region; POLR2A, RNA polymerase II; PWM, position weight matrix; TF, transcription factor; TSS, transcription start site.

Regarding the candidate genes, in case 44, a rare heterozygous missense variant [c.2500A > G, p.(Thr834Ala)] was identified in *ANO1*. Rare variants were identified in 2 probands (cases 38 and 49) within the 5′ regulatory region of the canonical *KCNK3* (*TASK-1*) transcript, in a putative regulatory island encompassing promoter elements and an epigenetically modified accessible region (EMAR) (53) (Fig. 2A). To further characterize these non-coding variants, their genomic positions were annotated using RegulomeDB v2.2 (GRCh38; Table S5) (30). The c.-75G > T variant (rs1315059704) received a RegulomeDB rank of 2c (score 0.72) and overlapped a predicted activator protein 2 (AP-2) family transcription factor biding motif (TFAP2A/B/C). The c.-17G > A variant (rs1364802137) received a RegulomeDB rank of 4 (score 0.61) and was located within the same active promoter element but did not overlap any predicted transcription factor binding motif (Fig. 2B).

In proband 4, the application of the allele-prioritization algorithm identified 2 VUS in the candidate genes *CASZ1* and *STRN*. Expression analysis in HAC15 cells showed that CASZ1 transcripts were barely detectable (*C*_t_ = 32 by qRT-PCR), indicating extremely low expression and precluding further functional studies, including gene silencing and variant-directed analyses (data not shown). Proband 4 also harbored a variant in the *STRN* gene, which encodes striatin, a ubiquitously expressed protein whose biological function remains incompletely characterized (Table 2).

To provide additional biological context for the prioritized candidate genes, we explored publicly available transcriptomic data from normal human adrenal tissue available in the GTEx database (Table S6) (30). *KCNK3* showed the highest transcript abundance among the evaluated genes (median TPM = 256.0), followed by *CACNA1H* (median TPM = 30.73), whereas *CASZ1* and *ANO1* exhibited comparatively lower expression levels (median TPM = 1.459 and 1.090, respectively).

### Genotype–phenotype correlations between carriers and noncarriers of rare germline variants

To explore genotype–phenotype correlations, patients were stratified into carriers of rare germline variants and those without detectable variants in the candidate genes. Baseline clinical, biochemical, histopathological, and imaging findings are summarized by group in Table 1. The cohort comprised 53 individuals, of whom 9 carried rare germline variants and 44 had no variants identified.

Notably, hypokalemia was markedly less frequent among variant carriers, occurring in only 1 of 9 (11%) compared with 17 of noncarriers (39.5%; *P* < .0001) (Table 1). In contrast, all variant carriers had resistant hypertension (9/9, 100%) vs 81.4% of noncarriers (35/44; *P* < .0001), and carriers required a greater antihypertensive treatment burden, with a median of 7 drugs (IQR 4.5-7.0) vs 4 (IQR 3.0-5.0; *P* = .0264).

Among patients with abnormal imaging, the pattern of laterality differed markedly between groups: bilateral involvement predominated in carriers (5/7, 71.4%) but was less common in noncarriers (9/25, 36.0%), whereas unilateral disease was the prevailing pattern in noncarriers (16/25, 64.0%) compared with carriers (2/7, 28.6%) (overall 18/32, 56.2% unilateral vs 14/32, 43.8% bilateral; *P* < .0001). A family history of hypertension was reported in 38 of 47 patients overall (80.9%) and was present in all variant carriers (8/8, 100%) vs 30 of 39 noncarriers (76.9%; *P* < .0001).

## Discussion

In the present study, we investigated germline variants associated with PA in a cohort of individuals with bilateral PA and resistant hypertension and/or hypertension diagnosed before 40 years of age, with apparently sporadic disease after excluding familial PA types 1 and 3. After applying the prioritization criteria, 9.6% of individuals in our cohort harbored variants in canonical genes (*CACNA1H*, *CACNA1D*), whereas 7.5% harbored variants in biologically plausible candidate genes (*ANO1*, *KCNK3*, *CASZ1*, *STRN*). Familial PA is a rare form of PA, accounting for less than 5% of bilateral cases, and the great majority of bilateral PA remains without a defined genetic basis. In contrast to the typical spectrum of familial PA, which is usually characterized by very early-onset hypertension (often before 20 years of age), our cohort showed clinical features more consistent with apparently sporadic PA. Interestingly, germline VUS in canonical genes, mostly in *CACNA1H*, were relatively frequent in this cohort of apparently sporadic bilateral PA.

Among the prioritized variants, the identification of alterations in *CACNA1H* and *CACNA1D* is particularly noteworthy given the established role of calcium signaling in adrenal aldosterone regulation. Both genes encode voltage-gated calcium channels expressed in zona glomerulosa cells and contribute to intracellular calcium influx, which represents a central mechanism controlling aldosterone biosynthesis through activation of calcium-dependent signaling pathways and stimulation of CYP11B2 expression (21, 54, 55). Recurrent germline variants in *CACNA1H* and *CACNA1D* act through a GoF mechanism and are established causes of Familial PA Type 4 (FH-IV | MIM #617027) and PASNA syndrome (Primary Aldosteronism, Seizures, and Neurologic Abnormalities | MIM #615474), respectively (21, 22).

To assess whether the *CACNA1x* variants identified here are concordant with a GoF mechanism, we applied funNCion, a machine-learning classifier developed specifically to predict the directional functional effect of missense variants in voltage-gated sodium and calcium channels (34). Among the 5 *CACNA1x* variants, only the *CACNA1H* p.(Val1553Ile) was predicted to be pathogenic and GoF; notably, this variant maps to the same domain III S6 segment, 4 residues C-terminal to p.(Met1549Val), whose GoF effect has been functionally established (22). The remaining variants were predicted to be functionally neutral, with no reliable directional assignment. This interpretation is consistent with reports that not all PA-associated *CACNA1H* variants are activating; for instance, the p.(Arg890His) variant exhibits loss-of-function behavior (56). Importantly, funNCion's training set included *CACNA1D* (GoF) variants in PA but did not include *CACNA1H*, whose predictions therefore rely on gene-family–level transfer; adrenal phenotypes were a small minority of the training data.

In this study, germline rare variants in *CACNA1H* were identified in 7.5% of individuals with apparently sporadic bilateral PA. Consistent with our findings, germline *CACNA1H* variants have been associated with early-onset PA, familial PA diagnosed between 30 and 40 years of age, and aldosteronoma with unilateral PA, suggesting that *CACNA1H* may act as a susceptibility gene predisposing to PA with diverse phenotypic presentations (57). Recently, we reported 3 germline VUS in *CACNA1H* associated with unilateral PA and classical histology (58). Taken together, these findings suggest that germline variants in *CACNA1H* may also be associated with bilateral PA, even in the absence of very early-onset disease, particularly in individuals with resistant hypertension or hypertension diagnosed before 40 years of age.

In addition to the variants discussed above, our analysis also identified rare alterations in other genes that may contribute to the genetic architecture of the disease. The *ANO1* gene, also known as *TMEM16A*, encodes a calcium-activated chloride channel belonging to the anoctamin family and plays a role in modulating intracellular calcium release through interactions with inositol 1,4,5-trisphosphate receptors (59). Cabrita et al (59) showed that *ANO1* overexpression enhances calcium release from intracellular stores following stimulation of G protein–coupled receptors, whereas reduced *ANO1* expression inhibits this process.

In a genome-wide association study, *CASZ1* has been mapped to a locus associated with PA (36). Several functional and molecular characterizations of this transcription factor have been reported, including its role as a co-repressor of mineralocorticoid receptor transcriptional activity (60). Among the regulatory targets of *CASZ1* is *CACNA1D* (49). In addition, reduced CASZ1 expression has been shown to promote aldosterone-dependent mineralocorticoid receptor transcriptional activity (60), whereas *CASZ1* overexpression suppresses aldosterone biosynthesis in adrenal cells (36). Although functional approaches such as exogenous overexpression and luciferase reporter assays could provide additional mechanistic insights into the impact of the identified variant, preliminary quantitative PCR analyses demonstrated very low endogenous *CASZ1* expression in human adrenocortical HAC15 cells, limiting the feasibility and interpretability of further functional experiments in this model.

In experiments using heterozygous *Strn* knockout mouse models, increased circulating aldosterone levels were observed, along with upregulation of renal mineralocorticoid receptor expression (61). In a complementary association study conducted by the same authors, risk alleles for salt-sensitive hypertension were also mapped to the *STRN* gene in a cohort of 366 Caucasian individuals (61). More recently, Stone et al reported the results of a clinical pharmacologic response protocol in patients with hypertension carrying 2 risk alleles in the *STRN* gene (rs2540923 or rs888083), demonstrating an excellent therapeutic response to eplerenone, a mineralocorticoid receptor antagonist, with substantial reductions in blood pressure levels (62). Evidence supporting a potential association between loss of function of the *STRN* gene and the pathogenesis of PA remains preliminary, and additional studies are required to further substantiate this relationship.

Interestingly, 2 VUS located in the regulatory region of the *KCNK3* (*TASK-1*) gene promoter were identified in our study. *KCNK3* encodes the TASK-1 potassium channel, which is highly expressed in the adrenal zona glomerulosa and plays an essential role in maintaining resting membrane potential and regulating aldosterone secretion (63). Genetic deletion or dysfunction of *TASK-1* in murine models disrupts background potassium conductance, leading to membrane depolarization, increased intracellular calcium influx, and autonomous aldosterone production independent of the renin–angiotensin-aldosterone system (64, 65).

In humans, *KCNK3* has been associated with a single clinical phenotype currently listed in OMIM (#615344), characterized by autosomal dominant pulmonary arterial hypertension with incomplete penetrance. Despite its established role in zona glomerulosa physiology, and although *KCNK3* polymorphisms have been associated with blood pressure regulation, hypertension, and plasma aldosterone levels in participants of the multi-ethnic study of atherosclerosis (66), no studies to date have directly linked *KCNK3* variants to adrenal disease in humans. Variants affecting regulatory regions and disrupting transcription factor binding have been reported in numerous monogenic disorders (67, 68). In this context, the *KCNK3* variants identified in our cohort (c.-17G > A p.? and c.-75G > T p.?) are particularly intriguing because they are located within an EMAR.

EMARs are genomic regions in which chromatin structure has been modified by epigenetic mechanisms, resulting in increased DNA accessibility to transcription factors, transcriptional machinery, and other regulatory complexes involved in gene expression control (69). Accessible chromatin regions frequently overlap functional regulatory elements, including promoters and enhancers, which play central roles in establishing cell-specific transcriptional programs and regulating gene expression (70). Therefore, although the identified *KCNK3* variants are located outside the protein-coding sequence, they may exert functional effects through regulatory mechanisms rather than structural alterations of the channel itself (71-73).

To move beyond region-level annotation and evaluate the regulatory potential of the variant positions, we annotated both variants using RegulomeDB v2.2, which integrates ENCODE transcription factor binding, chromatin accessibility, chromatin-state, and motif data. Both variants mapped to the same ENCODE cCRE, which overlaps a chromatin state classified as Active TSS in adrenal gland datasets, supported by chromatin accessibility, active promoter-associated histone marks (H3K4me3 and H3K27ac), and POLR2A/CTCF occupancy. The c.-75G > T variant (RegulomeDB rank 2c; score 0.72) overlapped a predicted AP-2 family transcription factor biding motif (TFAP2A/B/C), with TFAP2B ChIP-seq occupancy observed at this locus in the neuroblastoma cell line SK-N-SH. In adrenal tissue, POLR2A and CTCF occupancy further supported the regulatory activity of this promoter element in a disease-relevant context. By contrast, the c.-17G > A variant (RegulomeDB rank 4; score 0.61) was located within the same active promoter element but did not overlap any predicted transcription factor binding motif, indicating that the evidence supporting its regulatory relevance is primarily regional.

Together, these findings support the hypothesis that the upstream regulatory region of *KCNK3* is active in adrenal tissue and suggest that at least one of the identified variants, c.-75G > T, may alter transcription factor binding. This interpretation is biologically plausible given the established role of *KCNK3/TASK-1* in the electrophysiological regulation of zona glomerulosa cells and aldosterone production. The present analyses are computational and demonstrate overlap with regulatory features, including a predicted transcription factor binding motif disrupted by c.-75G > T, but do not establish allele-specific effects on transcription factor binding or *KCNK3* expression.

Interestingly, the present study identified relevant genotype–phenotype associations in patients with bilateral PA. Carriers of rare germline variants displayed a distinct clinical profile, including a lower frequency of hypokalemia, a markedly higher prevalence of family history of hypertension, and a predominance of tomographic abnormalities, particularly bilateral involvement. An interesting finding in our cohort was that individuals harboring biologically plausible candidate variants exhibited a higher frequency of resistant hypertension and required a greater antihypertensive treatment burden, suggesting a potentially more severe clinical presentation among variant-positive individuals. In addition, the greater frequency of bilateral adrenal abnormalities on computed tomography may reflect a more diffuse or genetically driven adrenal phenotype rather than a focal lesion.

In bilateral PA, adrenalectomy is generally not curative, as aldosterone excess arises from both glands. Surgery is therefore typically reserved for selected cases with lateralized or asymmetric disease, or for refractory hypertension with persistently suppressed renin. In a multicenter study, 43 patients with bilateral PA underwent unilateral adrenalectomy and 13 underwent bilateral sparing adrenalectomy (subtotal or total resection on one side combined with contralateral subtotal resection) (74). Among the 50 patients followed for 6 to 12 months, clinical benefit was observed in 30 of 37 (81%) after unilateral adrenalectomy and in 12 of 13 (92%) after bilateral sparing adrenalectomy.

This study has several limitations. First, the prioritized variants were not functionally validated and remain classified as VUS. This reflects the design of the study, which was conceived as a gene-discovery, hypothesis-generating effort aimed at identifying biologically plausible candidate genes. Reassuringly, the virtual panel was applied only after WES and variant filtering and was not guided by clinical features such as family history of hypertension, hypokalemia, or disease severity, reducing the likelihood of phenotype-driven associations. Nevertheless, the small number of variant-positive individuals limits the robustness of subgroup and genotype–phenotype analyses. A further limitation is the lack of formal segregation analysis, which constrains interpretation of inheritance patterns and variant contribution; recruitment of relatives and sample collection are already under way but could not be completed within the present timeframe. Confirmation in larger independent cohorts, together with functional assays, transcriptomic studies, and family-based replication, will be needed to clarify the biological consequences of these variants and their role in the pathogenesis of bilateral PA.

In conclusion, germline variants in canonical and candidate genes were identified in a subset of patients with apparently sporadic bilateral PA presenting with resistant or early-onset hypertension. Variants in established PA genes, particularly *CACNA1H*, were relatively frequent, and additional rare variants were detected in biologically plausible candidate genes, including *ANO1, KCNK3*, and *STRN*. Variant carriers displayed a distinct clinical profile, characterized by a lower frequency of hypokalemia, a higher prevalence of family history of hypertension, more frequent resistant hypertension with a greater antihypertensive treatment burden, and a predominance of bilateral adrenal abnormalities on imaging. Together, these findings suggest that germline variation may contribute to the genetic susceptibility and phenotypic heterogeneity of bilateral PA. Larger cohorts and integrated functional studies will be needed to establish the pathogenic relevance of these variants and their potential implications for the genetic evaluation and clinical management of affected patients.

## Data Availability

The data that support the findings of this study are available from the corresponding author upon reasonable request.

## Abbreviations

APA: aldosterone-producing adenoma
APM: aldosterone-producing micronodule
APN: aldosterone-producing nodule
AVS: adrenal venous sampling
CT: computed tomography
EMAR: epigenetically modified accessible region
GoF: gain of function
LoF: loss of function
PA: primary aldosteronism
TF: transcription factor
TPM: transcripts per million
VUS: variants of uncertain significance
WES: whole-exome sequencing
